# Supplementary material for: Sex-stratified pharmacovigilance of gastrointestinal events associated with first-line smoking-cessation medicines: Insights from the FAERS database
Source: PLoS One. 2025 Nov 6;20(11):e0336021. doi: 10.1371/journal.pone.0336021 (PMC12591404; doi:10.1371/journal.pone.0336021)
Supplement: S1 Table — (DOCX) [file pone.0336021.s001.docx]

**Supplementary material 1. Gastrointestinal (GI) Adverse Event Terms**

- Abdominal Discomfort
- Abdominal Distension
- Abdominal Pain
- Abdominal Pain Upper
- Anorexia
- Ascites
- Belching
- Bloating
- Bloody Stool
- Colitis
- Colitis Ischaemic
- Constipation
- Decreased Appetite
- Dental Caries
- Diarrhoea
- Dry Mouth
- Duodenal Ulcer
- Dyspepsia
- Enteritis
- Enterocolitis
- Epigastric Pain
- Esophagitis
- Flatulence
- Gastric Disorder
- Gastric Haemorrhage
- Gastric Perforation
- Gastric Ulcer
- Gastric Ulcer Haemorrhage
- Gastroenteritis
- Gastroesophageal Reflux
- Gastrointestinal Haemorrhage
- Gastrointestinal Perforation
- Gastrointestinal Toxicity
- Gingival Bleeding
- Gingival Ulceration
- Gingivitis
- Haematemesis
- Haematochezia
- Heartburn
- Hiccups
- Ileus
- Ileus Paralytic
- Immune-Mediated Enterocolitis
- Intestinal Disorder
- Intestinal Ischaemia
- Intestinal Obstruction
- Intestinal Perforation
- Intra-Abdominal Fluid Collection
- Loss Of Appetite
- Mechanical Ileus
- Melena
- Mouth Ulceration
- Nausea
- Neutropenic Colitis
- Oesophageal Varices Haemorrhage
- Oral Disorder
- Oral Pain
- Pancreatitis
- Pancreatitis Acute
- Peptic Ulcer
- Periodontal Disease
- Ptyalism
- Rectal Bleeding
- Salivary Hypersecretion
- Small Intestinal Obstruction
- Small Intestinal Perforation
- Stomach Pain
- Stomatitis
- Taste Alteration
- Tongue Ulceration
- Upper Gastrointestinal Haemorrhage
- Varices Oesophageal
- Vomiting
- Xerostomia
