# Supplementary material for: Sex-stratified pharmacovigilance of gastrointestinal events associated with first-line smoking-cessation medicines: Insights from the FAERS database
Source: PLoS One. 2025 Nov 6;20(11):e0336021. doi: 10.1371/journal.pone.0336021 (PMC12591404; doi:10.1371/journal.pone.0336021)
Supplement: Attachment — Submitted filename: Response to reviewer #2.docx [file pone.0336021.s004.docx]

We are grateful to Reviewer #2 for their thorough and detailed feedback, which has led to substantial improvements throughout the manuscript. We have addressed all points raised below.

**Comment 1:** *“Throughout the abstract, Key points, plain language summary, and main text, the authors made a lot of statements about the prevalence of side effects... Please note that there is no denominator... All of the statements... should be made on the adverse event reports, rather than the overall drug use to avoid reporting bias...”*

**Response:** This is an essential point regarding the limitations of FAERS data. We have carefully revised the entire manuscript (including the Abstract, Key Points, and Plain Language Summary) to ensure our language is precise.

We now consistently use terms such as “reporting profiles,” “disproportionality signals,” and “proportion of reports” instead of terms that might imply prevalence or causal risk (e.g., “prevalence,” “risk,” or “produced”).

**(See revisions throughout the Abstract, Results, and Discussion sections)**

**Comment 2:** *“In the Introduction, the authors mentioned ‘the comparative risk profiles... have not been comprehensively quantified...’ Could the authors add 1-2 sentences to summarize the findings from published literature? ...a strong rationale is needed to justify why FAERS data was used.”*

**Response:** We thank the reviewer for this suggestion. We have revised the Introduction to strengthen our rationale. We now explicitly state the limitations of controlled trials and other observational studies (e.g., their inability to detect rare signals or reflect real-world heterogeneity) to better justify the unique value of a large-scale pharmacovigilance study using the FAERS database for hypothesis generation.

**(See Introduction)**

**Comment 3:** *“‘The findings provide clinicians with an evidence-based appraisal of relative GI risk...’ This may be an overstatement with the limitations of FAERS data. Safety signals from FAERS do not imply and should not be interpreted as risk to guide decision making.”*

**Response:** We agree that our original statement was an overstatement. We have toned down the language in the Discussion and Conclusion to reflect the hypothesis-generating nature of our findings. We now state that these results cannot establish causality or directly guide clinical practice but can highlight areas for future research and inform clinical consideration.

**(See Discussion and Conclusion)**

**Comment 4:** *“Please describe the methods of identifying reports containing smoking cessation medications (ie, search terms) from FAERS. A summary table is preferred.”*

**Response:** As requested, we have added **Table 1**, which provides a comprehensive list of the search terms (both generic and brand names) used to identify reports for NRT, varenicline, and bupropion in the FAERS database.

**(See Methods section and Table 1)**

**Comment 5:** *“‘A signal was considered present if a given drug–event (DE) pair had at least three reports and met the threshold of PRR ≥ 2 with an accompanying chi-square (χ²) value ≥ 4.’ Please add a reference for this statement.”*

**Response:** We have now added a citation for this established signal detection criterion in pharmacovigilance.

**(See Methods section, "Data Mining" subsection)**

**Comment 6 & 9:** *“The authors wanted to explore the sex-stratified safety signal, but there is no mention of methodology or statistical analysis. Please clarify”* and *“For comparing ROR/PRR between male and female, a statistical test may be needed...”*

**Response:** We apologize for this critical omission. As also noted by Reviewer #1, we have now added a detailed methodology for the sex-stratified analysis. A new subsection, "**Sex-stratified disproportionality and Breslow–Day test**," has been added to the Methods section. This section describes how we performed the stratification and used the Breslow–Day test to formally assess for statistically significant differences in reporting between sexes. The results are presented in **Table 4** and **Figure 3**.

**(See Methods section, and Results section, Table 4 and Figure 3)**

**Comment 7:** *“In the results, please add a flowchart diagram describing the steps of obtaining the final FAERS reports...”*

**Response:** We thank the reviewer for this suggestion to improve clarity. We have added a flowchart (**Figure 1**) that visually details the entire process of case selection, from the initial number of reports in the database to the final dataset used in our analysis.

**(See Results section, Figure 1)**

**Comment 8:** *“Please add counts of reports along with the % for descriptive statistics.”*

**Response:** We have revised our descriptive statistics to include both absolute counts (n) and percentages (%). This format has been applied to the main text of the Results section and is clearly presented in **Table 2**, where the header clarifies that values are "n (% of all reports for that drug)."

**(See Results section and Table 2)**
